# AI-Assisted Clinical Data Abstraction From Electronic Health Records: Retrospective Concordance Study

**DOI:** 10.2196/96755

**Published:** 2026-07-07

**Authors:** Camille Sarah Schwartz, Michael John Anderson, Kelsey Nicole Moakler, Bradley Adam Newby, David Alan Davenport, Matthew Wilson Schwartz

**Affiliations:** 1 University of Nevada, Reno, 1664 N Virginia St, Reno, NV, 89557, United States, 1 7027529240; 2 Comprehensive Cancer Centers of Nevada (CCCN), Henderson, NV, United States; 3 Kirk Kerkorian School of Medicine, University of Nevada, Las Vegas, Las Vegas, NV, United States

**Keywords:** medical informatics, natural language processing, electronic health records, clinical data abstraction, artificial intelligence, AI, large language models

## Abstract

**Background:**

Manual chart abstraction from electronic health records is a critical step in clinical outcomes research but is time-intensive and prone to human error. Advances in artificial intelligence (AI), particularly large language models, offer the potential to automate the extraction of structured data from unstructured clinical documentation with improved efficiency and consistency.

**Objective:**

This study aimed to evaluate the accuracy and efficiency of an AI-assisted approach for extracting patient-reported outcomes from clinical notes compared with traditional human abstraction.

**Methods:**

We conducted a retrospective study of 26 patients treated with low-dose radiation therapy for osteoarthritis. Human reviewers abstracted numeric rating scale (NRS; 0‐10) pain scores at baseline, the end of treatment, and the first follow-up, and von Pannewitz score (VPS; 0‐4) improvement scores at posttreatment time points. A HIPAA (Health Insurance Portability and Accountability Act)–compliant generative pretrained transformer–based AI system was prompted to extract the same end points from clinical notes. Concordance was assessed using exact match rates, the intraclass correlation coefficient for the NRS, and weighted Cohen κ for the VPS. The time required for AI vs manual abstraction was recorded. The AI system was not trained or fine-tuned on study data, and performance was evaluated directly against human abstraction to reflect real-world deployment.

**Results:**

The AI system demonstrated high concordance with human abstraction, achieving an exact match rate of 92% for the NRS (95% CI 84‐96; intraclass correlation coefficient=0.96) and 94% for the VPS (95% CI 84‐98; κ=0.91). All discrepancies were minor, and no spurious values were generated. The AI system identified 1 clinically relevant data point missed during manual review. Average abstraction time per patient decreased from approximately 30 minutes to 2 minutes, representing time savings of >90%. The system also captured trends in analgesic use, but these results were not statistically significant, including reductions without escalation.

**Conclusions:**

AI-assisted data abstraction demonstrated high concordance with human review in this single-institution cohort while substantially reducing the time requirements. These findings support the feasibility of AI-assisted abstraction workflows, although further validation across larger and more diverse datasets is needed.

## Introduction

Extraction of structured clinical data from electronic health records (EHRs) remains a major bottleneck in clinical research, requiring substantial time and being prone to human error. These inefficiencies highlight the need for more efficient and reproducible approaches to extracting structured data from clinical documentation. For example, Johns Hopkins Hospital alone spends more than US $5 million annually on quality reporting, with human data abstraction being a major driver of that cost [1]. Clinicians also spend significant time per encounter reviewing charts, representing an enormous system-wide burden.

Osteoarthritis is a prevalent degenerative joint disease affecting more than 30 million adults in the United States and often causing chronic pain and disability [2]. Management is typically conservative (eg, analgesics and physical therapy) until advanced cases warrant surgery. However, many patients experience inadequate relief or are not surgical candidates. There is growing interest in intermediate therapies that can alleviate pain without the risks associated with long-term medications or invasive procedures.

Low-dose radiation therapy (LDRT) has re-emerged as a potential noninvasive treatment for osteoarthritis pain. Historically used in parts of Europe for benign conditions such as arthritis, LDRT involves delivering very low radiation doses to affected joints. Modern series have reported pain improvement rates of approximately 50% to 80% after LDRT (typically approximately 3 Gy in 6 fractions) [3]. A recent US multi-institutional retrospective study found that 84% of treated patients experienced pain relief with LDRT [4]. Similarly, an interim analysis of a randomized trial showed significantly greater pain improvement (approximately 70% of patients) with LDRT than with sham treatment (approximately 42%) [5]. These findings suggest that LDRT can provide meaningful analgesia for osteoarthritis, potentially filling a treatment gap between pharmacologic management and surgery.

As interest in LDRT grows, rigorous outcomes research is needed to characterize its efficacy and safety. Retrospective chart reviews are a common approach to evaluate real-world outcomes, but manual chart abstraction is notoriously time-consuming and prone to error. Extracting pain scores, functional outcomes, and medication changes from free-text clinical notes can take many hours, even for modest sample sizes. Moreover, human abstraction is subject to interobserver variability; different reviewers may interpret or transcribe data inconsistently. This poses a challenge for scaling up multi-institutional studies or registries to assess LDRT outcomes.

Advances in natural language processing (NLP) and artificial intelligence (AI) offer a potential solution. Transformer-based large language models (LLMs), such as generative pretrained transformer (GPT)–based systems, can comprehend and summarize unstructured text with remarkable proficiency. In other medical domains, NLP algorithms have demonstrated high accuracy in extracting clinically relevant information from unstructured EHRs when compared with human chart review [6-8]. Integrating AI into data abstraction could greatly improve efficiency and consistency, enabling larger real-world evidence studies. However, the performance of general-purpose LLMs for detailed clinical data abstraction tasks remains to be validated.

In this study, we applied a HIPAA (Health Insurance Portability and Accountability Act)–compliant AI assistant (Microsoft Copilot, a GPT-based LLM) to extract key outcome variables from the medical records of patients with osteoarthritis treated with LDRT at our institution. We then compared the AI system’s outputs with those obtained through traditional human abstraction. The primary objective was to assess concordance between AI and human reviewers in capturing pain scores and patient-reported improvements. Secondary objectives included comparing the time efficiency of AI with that of manual extraction, characterizing any discrepancies to identify error patterns, and exploring the AI system’s ability to capture analgesic use trends. In addition to evaluating clinical outcomes, this study focused on validating an AI-assisted approach for automated data abstraction from unstructured clinical documentation under real-world conditions.

## Methods

### Patient Cohort and Treatment

We retrospectively analyzed 26 patients with peripheral joint osteoarthritis who were treated with LDRT at a single academic center from 2022 to 2024. All patients had chronic, painful osteoarthritis (confirmed on imaging) that was refractory to conventional therapy (eg, nonsteroidal anti-inflammatory drugs and injections) and were referred for LDRT as an alternative. LDRT was delivered using 6- or 18-MV photons to the affected joint(s) at a dose of 0.5 Gy per fraction, given 2 to 3 times per week for a total of 6 fractions (total dose of 3.0 Gy). This regimen, based on German protocols and prior literature [3], was chosen to balance pain relief and minimal toxicity. Treatments were delivered on standard linear accelerators without anesthesia or sedation.

We compiled the relevant clinical notes for each patient at 3 key time points: baseline (the initial consultation note before or at the start of LDRT), end of treatment (EOT; the radiation treatment completion note, approximately 2 to 3 weeks after treatment initiation), and first follow-up (the first posttreatment follow-up visit, typically 4 to 6 weeks after LDRT). These notes, authored by attending radiation oncologists, documented pain levels, patients’ subjective improvement, and any changes in medications or function. The aggregated dataset comprised approximately 110 notes (approximately 250 pages of text and approximately 90,000 words) of unstructured clinical text, reflecting the complexity of real-world documentation.

Although pain scores are numeric, they were embedded within free-text clinical narratives with variable syntax, placement, and phrasing (eg, within structured fields, dictated prose, or assessment text). No standardized template was used across notes, reflecting routine radiation oncology documentation. Although medication extraction was not explicitly defined as a structured end point in the prompt, the model’s ability to summarize relevant clinical context enabled the identification of medication-related observations, which were analyzed qualitatively. This study therefore evaluates AI performance under real-world clinical documentation conditions rather than using curated or fully structured inputs.

### Data Abstraction: Human Review

Two independent reviewers performed manual data abstraction. Reviewers worked independently, and discrepancies were resolved through consensus discussion to generate the reference dataset. For manual data abstraction, investigators reviewed all compiled notes and recorded the numeric rating scale (NRS) and von Pannewitz score (VPS) values at each time point (baseline, EOT, and follow-up) for each patient. Any ambiguities or discrepancies in the chart data were resolved through discussion, yielding a consensus “gold standard” dataset of pain and improvement scores for all patients.

Human reviewers were not exposed to the AI system’s outputs during initial abstraction. Discrepancies were resolved through joint review to establish a consensus reference dataset, reflecting standard practice in retrospective outcomes research.

In addition to pain and improvement scores, the human reviewers noted any qualitative descriptions of pain relief and changes in analgesic medication use between baseline and follow-up to provide context for the outcomes.

### Data Abstraction: AI Model

For AI-driven abstraction, we used Microsoft Copilot in a HIPAA-compliant enterprise environment. The AI system was provided with concatenated clinical notes for each patient, clearly delineated by time point (baseline, EOT, and first follow-up). A structured, constraint-based prompt was used to specify predefined end points (NRS and VPS), fixed clinical time points, and explicit instructions to return “Not documented” when values were absent or ambiguous, rather than inferring or imputing data. The model was accessed via Microsoft Copilot between August 18, 2025, and August 22, 2025. A fixed prompt was used for all cases without modification. AI system outputs were not modified prior to analysis, and all accuracy metrics were calculated using the raw AI-generated outputs compared with the human consensus reference standard.

If multiple scores were present within a note, the AI system was instructed to select the clearest physician- or patient-reported numeric value, mirroring standard human abstraction practices. Each extracted value was required to be accompanied by a supporting text snippet from the source note, enabling direct traceability and verification. This design was intended to minimize the risk of hallucination and to ensure that AI system outputs remained auditable against the original clinical documentation.

This prompting strategy parallels the approach described by Dagli et al [9], who used a 2-stage NLP+GPT-4 pipeline to constrain token use and reduce hallucinations in an automated operative note abstraction system, achieving near-perfect accuracy. By similarly constraining the AI system’s task and instructing it to avoid making up data, we aimed to minimize the risk of hallucinated values.

After processing each patient’s notes, the AI system produced a structured output with the extracted NRS scores and VPS for each time point and a summary of any medication changes. These outputs were then compared with the human-derived dataset. AI system outputs were reviewed for completeness but were not corrected prior to analysis. No clinical decisions were made based on AI-generated data.

This study did not involve model training, fine-tuning, or prompt optimization using outcome data. A single, prespecified prompt was used for all cases and was not modified based on performance. Because the AI system was a fixed, general-purpose model, traditional training or validation splits were not applicable; rather, performance was evaluated directly against human abstraction, which reflects the real-world comparator that this workflow is intended to replace. Although medication extraction was not explicitly defined as a primary structured end point in the prompt, the model’s ability to summarize relevant clinical context allowed the identification of medication-related changes, which were analyzed qualitatively.

### Comparison Metrics and Statistical Analysis

We compared the AI-extracted data to the human consensus reference for each time point and patient. Concordance was first assessed using the exact match rate—the percentage of data points for which the AI-generated value exactly matched the human-derived value. Discrepancies were categorized as *minor* (eg, a small numeric difference likely attributable to rounding or a missed borderline mention) or *major* (eg, a clearly incorrect or spurious value).

We further quantified agreement using statistical measures. For the continuous NRS (0‐10) pain scores, we computed a 2-way mixed-effects intraclass correlation coefficient (absolute agreement) between AI and human readings. For the ordinal VPS (0‐4 improvement scale), we calculated a linear weighted Cohen κ to assess agreement. We also specifically noted whether the AI system identified any data points that the human reviewers missed, or vice versa, to determine whether AI could augment human abstraction.

The time required for data abstraction by the human reviewers (in total hours and per patient) was recorded prospectively. Similarly, we recorded the AI system processing time per patient, including the time required for prompting and light verification of outputs, reflecting real-world workflow conditions.

Basic descriptive statistics were used to summarize differences in abstraction time. We performed a paired 2-tailed *t* test to evaluate changes in NRS pain scores from baseline to first follow-up. Mean change, SD, and corresponding *P* values were calculated. 95% CIs were computed for all primary agreement metrics, including exact match rates and weighted Cohen κ, to account for the small sample size and provide estimates of precision. We summarized analgesic use trends qualitatively. Statistical analyses were conducted using standard software, with a significance threshold of *P*<.05 for hypothesis tests. The selected agreement metrics were chosen a priori based on the scale properties of each outcome (continuous NRS and ordinal VPS).

Multimedia Appendix 1 provides the full AI abstraction prompt used in this study.

### Ethical Considerations

This retrospective study was reviewed and approved by the institutional review board of the University of Nevada, Las Vegas (UNLV-2025-460). The study was determined to meet the criteria for exempt research involving secondary analysis of existing clinical data. The AI-assisted abstraction analysis was conducted as a secondary evaluation within this institutional review board–approved dataset.

A full waiver of HIPAA authorization was granted due to the retrospective nature of the study and the minimal risk posed to participants. Patient data were accessed solely for cohort identification and data abstraction purposes. No direct identifiers were recorded in the study dataset, and all data were deidentified prior to analysis.

All data were handled in accordance with institutional policies and applicable privacy regulations. Data were stored on secure, password-protected systems accessible only to authorized study personnel. No identifiable patient information is reported in this study.

This study was conducted as a retrospective feasibility assessment of AI-assisted data abstraction rather than as a predictive modeling study. Accordingly, standard reporting guidelines such as TRIPOD (Transparent Reporting of a Multivariable Prediction Model for Individual Prognosis or Diagnosis)-AI or CONSORT (Consolidated Standards of Reporting Trials)-AI were not fully applicable. However, key principles from these frameworks were incorporated, including clear definitions of inputs and outputs, transparent reporting of model use, and specification of performance metrics and limitations.

## Results

### Concordance of AI vs Human Abstraction

#### Pain Scores (NRS)

Out of 78 total NRS entries (26 patients×3 time points), the AI-extracted pain score exactly matched the human-abstracted score in 72 instances, yielding an exact match rate of 92.3% (95% CI 84.2‐96.4). The 6 mismatches were all minor (differences of 1‐2 points). There were no major errors in which the AI system produced an implausible or clearly incorrect pain score. The correlation between AI-extracted and human-extracted NRS values was excellent, with an intraclass correlation coefficient of 0.96 (95% CI 0.93‐0.98), indicating very high agreement.

Mean NRS pain scores decreased significantly from baseline to first follow-up (paired 2-tailed *t* test: *P*<.001), supporting a statistically significant improvement in patient-reported pain following treatment.

#### Improvement Scores (VPS)

Among 52 applicable VPS entries (26 patients×2 postbaseline time points, as improvement is not assessed at baseline), the AI system exactly matched the human value in 49 cases, yielding an exact match rate of 94.2% (95% CI 84.1-98.1). The 3 discrepancies were minor (differences of 1 category of improvement). No major discrepancies were observed for the improvement scores. Agreement was very high, with a weighted κ of 0.91 (95% CI 0.80-1.00), consistent with very high agreement between AI and human reviewers. Table 1 summarizes concordance between AI and human abstraction across all outcome measures.

**Table 1.** Concordance between artificial intelligence (AI) and human data abstraction (N=26).

Outcome metrics	Data points	Exact matches (AI vs human), n (%; 95% CI)	Minor differences, n (%)	Major differences, n (%)	Agreement statistic (95% CI)
Numeric rating scale (10-point pain scale)	78	72 (92.3; 84.2‐96.4)	6 (7.7)	0 (0)	0.96^a^ (0.93‐0.98)
von Pannewitz score (4-point improvement scale)	52	49 (94.2; 84.1‐98.1)	3 (5.8)	0 (0)	0.91^b^ (0.80‐1.00)

a Intraclass correlation coefficient.

b Weighted κ.

Notably, the AI system did not introduce any false data points that were not present in the notes. In fact, the AI system identified 1 pain score that the human reviewers had initially missed in the narrative text (a follow-up note in which the pain score was mentioned in a less-structured way). This finding suggests that the AI system may serve as a safety net for human oversight in some cases. There were no instances in which the AI system “hallucinated” a value that was not documented in the charts.

Overall, the AI-extracted values showed high agreement with the human consensus reference. All observed discrepancies were minor and did not alter the interpretation of clinical outcomes (eg, an NRS difference of 1-2 points or adjacent VPS categories). No instances of spurious or hallucinated values were observed. Importantly, there were no cases of major errors that would mislead conclusions (ie, no completely missed pain documentation or invented scores by the AI system).

### Data Extraction Efficiency

In addition to demonstrating high accuracy, the AI method showed a dramatic improvement in speed. Manual abstraction by the human reviewers required approximately 12 to 14 hours in total for 26 patients (approximately 30 min per patient on average, considering the need to read through notes carefully and double-check values). In contrast, AI-based abstraction, including the time required to prompt the model and lightly verify the outputs, was completed in <1 hour for all 26 patients (approximately 2‐3 min per patient for processing, plus a few s of oversight per case). This corresponds to approximately a 10- to 15-fold increase in speed for data extraction using AI compared to the traditional manual approach. In practical terms, what took a human several workdays to accomplish was completed by the AI system in approximately 1 hour without loss of fidelity.

### Pain and Medication Outcomes

In terms of analgesic use, the AI system successfully captured notes about medication changes in 92.3% (n=24) of 26 cases (the remaining 2, 7.7% patients had no mention of medications in the notes). Of those 24 patients, 33.3% (n=8) were able to reduce or discontinue some pain medications by the first follow-up (eg, by decreasing nonsteroidal anti-inflammatory drug use or discontinuing opioids), reflecting clinical improvement. The remaining patients had no change in analgesic regimen, and no cases of escalation in pain medication use were noted during the short follow-up period.

## Discussion

### Principal Findings

Manual chart abstraction has long been recognized as a bottleneck in outcomes research. It is labor intensive, slow, prone to inconsistent accuracy, and costly at scale. Recent commentaries have argued that the traditional approach of clinicians poring over charts is becoming unsustainable [1]. Major academic centers spend millions on manual data reporting, and physicians devote substantial time to record reviews that could be better spent on patient care. These inefficiencies create a strong motivation to explore more efficient and sustainable alternatives. The novelty of this work lies not in algorithm development, but in demonstrating that a constrained, auditable AI workflow can approximate a common, resource-intensive manual abstraction task with high agreement in a defined clinical setting. Rather than replacing human review, this approach may serve as a complementary tool to reduce manual workload while maintaining accuracy.

Our study contributes to a growing body of evidence that AI can help address this challenge. Using an LLM deployed in a HIPAA-compliant clinical environment, we demonstrated that AI-based abstraction of clinical notes can achieve high agreement with human reviewers, while reducing the time burden by more than 90%. The AI system in our study even identified an outcome data point missed by human reviewers, highlighting its potential as a quality assurance aid. These findings suggest that, for well-defined and repetitive abstraction tasks, AI-assisted workflows can achieve performance comparable to human review while offering substantial efficiency gains. Human oversight remains essential for contextual interpretation and validation, but AI may meaningfully reduce the manual burden associated with large-scale outcomes research.

Our findings closely align with a growing body of literature demonstrating that LLMs and NLP systems can accurately extract structured clinical information from unstructured EHRs [10,11]. Dagli et al [9] validated an NLP- and LLM-based framework for automated chart review and reported high extraction accuracy with substantial reductions in manual effort. Similarly, Ntinopoulos et al [12] demonstrated strong performance across multiple LLMs for the extraction of structured information from unstructured and semistructured EHRs. Similar findings have been reported for the extraction of social determinants of health from clinical notes across institutions using LLMs [13]. Large clinical language models trained specifically on EHR data, such as GatorTron, have further demonstrated substantial improvements across multiple clinical NLP tasks, supporting the feasibility of AI-assisted extraction from complex clinical documentation [14].

Our results are also consistent with emerging oncology-specific evidence [15]. Chen et al [16] conducted a scoping review of LLM applications for health information extraction in oncology and concluded that LLMs show considerable promise for extracting clinically relevant information from unstructured oncology data sources while improving research efficiency and scalability. Kang et al [17] recently reported that LLM-assisted curation of breast cancer clinical data achieved high agreement with manual review while substantially reducing abstraction time. Similarly, Bilal et al [18] reviewed 94 cancer-focused NLP studies and identified information extraction as the most common application, highlighting the growing role of transformer-based models in oncology research and outcomes assessment. A recent systematic review by Dahl et al [19] similarly demonstrated strong performance of NLP systems for information extraction from cancer-related EHRs. Collectively, these findings support the growing role of AI-assisted workflows in clinical research and suggest that appropriately constrained LLM-based abstraction systems may substantially reduce manual workload while maintaining high levels of accuracy.

The growing adoption of AI-assisted data extraction extends across multiple clinical specialties. Farrow et al [20] identified expanding applications of NLP in trauma and orthopedics, highlighting the potential of these technologies to improve data capture, outcomes assessment, and research efficiency from routinely collected clinical documentation. Within radiation oncology specifically, Bitterman et al [21] described numerous opportunities for clinical NLP to support outcomes research, toxicity assessment, quality improvement initiatives, and automated data extraction from clinical records. These findings suggest that AI-assisted abstraction may have broad applicability across diverse clinical research settings.

Recent guidance documents have emphasized the importance of transparency, accountability, and human oversight when implementing AI systems in health care. The TRIPOD+AI, CONSORT-AI, and SPIRIT (Standard Protocol Items: Recommendations for Interventional Trials)-AI reporting frameworks provide recommendations for transparent reporting of AI-based research and clinical applications [22-24]. Similarly, the World Health Organization has highlighted the importance of governance, auditability, and ongoing human supervision when deploying large multimodal models in health care environments [25]. Consistent with these recommendations, our study used a constrained and auditable abstraction workflow in which all AI-generated outputs were reviewed against source documentation and compared with a human consensus reference standard.

It is important to note the limitations of our study. We examined a relatively small sample (N=26) at a single institution, focusing on a narrow set of end points (pain and improvement scores) in 1 disease context. The generalizability of GPT-based AI-assisted abstraction to other contexts (eg, different document types, outcome variables such as toxicity grades or laboratory results, and different clinical specialties) needs further study. Additionally, although our AI system showed no inclination to hallucinate data in this constrained task, more complex abstraction tasks might pose greater challenges. We also manually reviewed the AI system’s outputs in this study; a fully autonomous system would require robust validation and likely periodic auditing. Additionally, human abstraction itself is subject to interobserver variability, and the consensus reference used here represents a practical comparator rather than an absolute ground truth. Prior studies have similarly demonstrated variability among human reviewers during manual chart abstraction, highlighting that the reference standard itself is subject to measurement error and may not represent an absolute ground truth. CIs were reported for all agreement metrics to reflect the statistical uncertainty associated with the small sample size. This study was conducted at a single academic institution using a relatively small cohort (N=26). Clinical documentation styles vary across institutions, and model performance may differ in other settings. Accordingly, these findings should be interpreted as a feasibility assessment rather than evidence of generalizable performance.

Future work should explore scaling this approach across multiple institutions to ensure that the AI system maintains performance with more heterogeneous data. It would also be informative to integrate AI-based abstraction into a live clinical workflow—for example, by continuously populating a database from clinic notes—and measure real-world efficiency gains and any issues that arise. Performing a cost-benefit analysis (considering the time saved against the resources needed to implement the AI system) would help quantify the value proposition. As LLMs continue to advance, specialized fine-tuning for medical text extraction could further improve robustness and consistency across more complex abstraction tasks.

In summary, our experience suggests that tasks that have traditionally required tedious manual effort in retrospective studies may be supported by AI-assisted workflows. We provide evidence that AI-driven chart review can match human abstraction accuracy while substantially reducing time requirements. The use of AI-assisted abstraction may help reduce the manual burden associated with retrospective data collection and support more efficient research workflows.

Importantly, we view AI not as a replacement for human expertise but as an augmentation tool. In our study, human reviewers defined the data points, validated outputs, and interpreted their significance—roles that remain crucial. The AI system handled the routine extraction. This synergy can enhance the productivity of research teams. With appropriate oversight, AI-assisted workflows have the potential to support more efficient approaches to outcomes research.

### Conclusions

This pilot feasibility study demonstrates that AI-assisted clinical data abstraction can approximate manual chart review with high accuracy while substantially improving efficiency. In a cohort of patients with osteoarthritis treated with low-dose radiotherapy, an AI model (Microsoft Copilot) demonstrated high concordance with human data abstraction while reducing the time required for abstraction by more than 90%. No significant errors were introduced by the AI system, and it was capable of identifying a detail missed by human reviewers, illustrating a potential complementary role in ensuring data completeness.

These findings suggest that AI-assisted approaches may play a role in addressing increasing data abstraction demands in clinical research [1]. By validating the AI system against a human gold standard and clearly reporting performance metrics, we aim to provide a framework for responsible adoption of this technology in radiation oncology. Wider implementation of AI-assisted abstraction could streamline multi-institution studies, registries, and routine quality reporting, allowing researchers and clinicians to obtain insights more rapidly and consistently.

Such tools may improve efficiency while maintaining data accuracy and could support more efficient clinical research workflows.

## Supplementary material

10.2196/96755 Multimedia Appendix 1 Artificial intelligence system abstraction prompt used for extraction of pain outcomes.

## Abbreviations

AI: artificial intelligence
CONSORT: Consolidated Standards of Reporting Trials
EHR: electronic health record
EOT: end of treatment
GPT: generative pretrained transformer
HIPAA: Health Insurance Portability and Accountability Act
LDRT: low-dose radiation therapy
LLM: large language model
NLP: natural language processing
NRS: numeric rating scale
SPIRIT: Standard Protocol Items: Recommendations for Interventional Trials
TRIPOD: Transparent Reporting of a Multivariable Prediction Model for Individual Prognosis or Diagnosis
VPS: von Pannewitz score
