# Supplementary material for: AI-Assisted Clinical Data Abstraction From Electronic Health Records: Retrospective Concordance Study
Source: JMIR Form Res. 2026 Jul 7;10:e96755. doi: 10.2196/96755 (PMC13340080; doi:10.2196/96755)
Supplement: Multimedia Appendix 1 [file formative-v10-e96755-s001.docx]

Supplementary Appendix A

AI Abstraction Prompt Used for Extraction of Pain Outcomes

Purpose

This appendix provides the full prompt used to guide the artificial intelligence (AI) system in extracting patient-reported pain and improvement outcomes from unstructured clinical notes. The prompt was designed to constrain the AI’s task, minimize hallucination risk, and ensure traceability of all extracted values to source documentation.

AI System

Microsoft Copilot (GPT-based large language model) deployed in a HIPAA-compliant enterprise environment.

Prompt Text (As Used in the Study)

Task:
Review the following patient clinical note(s) and extract all available Numeric Rating Scale (NRS) pain scores (0–10) and von Pannewitz Score (VPS) patient-assessed improvement scores (0–4).

Instructions:

1. Identify NRS pain scores (0–10) recorded at the following timepoints:
   - Baseline (before treatment)
   - End of Treatment (EOT)
   - Approximately 1-month follow-up (“Pre-90” note)
2. Identify VPS improvement scores (0–4) recorded at:
   - End of Treatment (EOT)
   - Approximately 1-month follow-up (“Pre-90” note)
3. If multiple scores are present within a note or timepoint, select the clearest physician- or patient-reported numeric value.
4. If no score is found for a given timepoint or metric, label the field as “Not documented.”
   - Do not infer, estimate, or impute values.
5. Ignore unrelated historical information and focus only on documentation relevant to the specified timepoints and outcomes.

Required Output Format (Structured Table):

| Patient ID | Date | Timepoint (Baseline / EOT / Follow-up) | NRS Score (0–10) | VPS Score (0–4) | Supporting Text Snippet |

Each extracted value must be accompanied by a supporting text snippet quoted directly from the source note to allow verification and auditability.

Example Output:

| Patient ID | Date | Timepoint | NRS Score | VPS Score | Supporting Text Snippet |
| --- | --- | --- | --- | --- | --- |
| 12345 | 2025-01-22 | Baseline | 8 | Not documented | “Patient reports pain 8/10 prior to starting LDRT.” |
| 12345 | 2025-02-10 | EOT | 5 | 2 | “EOT summary note: pain improved, VPS = 2.” |
| 12345 | 2025-03-12 | Follow-up | 3 | 1 | “Pre-90 visit note: pain 3/10, patient states significant improvement.” |

Rationale

This prompt was intentionally constrained to:

- Predefined outcome instruments (NRS, VPS)
- Fixed clinical timepoints
- Explicit handling of missing data (“Not documented”)
- Mandatory provenance via supporting text snippets

These constraints were implemented to reduce hallucination risk, prevent inference beyond documented data, and ensure direct traceability between AI-extracted values and original clinical documentation.
